# Di-μ_2_-chlorido-bis­[chlorido(η^6^-hexa­methyl­benzene)ruthenium(II)]

**DOI:** 10.1107/S1600536809041154

**Published:** 2009-10-17

**Authors:** Yunuem González-Torres, Noel Espinosa-Jalapa, Simón Hernández-Ortega, Ronan Le Lagadec, David Morales-Morales

**Affiliations:** a Instituto de Química, Universidad Nacional Autónoma de México, Circuito Exterior, Ciudad Universitaria, México 04510, Mexico

## Abstract

Dimeric mol­ecules of the title compound, [Ru_2_Cl_4_(C_12_H_18_)_2_], are located on a crystallographic centre of inversion with one mol­ecule in the asymmetric unit. The hexa­methyl­benzene rings are in an η^6^-coordination to the ruthenium centres, which are bridged by two chloride ligands. In addition, the ruthenium centres are bonded to another chloride ligand. The aromatic rings and the Ru_2_Cl_2_ four-membered ring enclose a dihedral angle of 55.85 (6)°.

## Related literature

For the properties and potential applications of half-sandwich ruthenium (II) complexes, see: Le Bozec *et al.* (1989 ▶); Leyva *et al.* (2007 ▶); Ryabov *et al.* (2001 ▶). For our work on the synthesis and catalytic applications of different ruthenium–arene complexes, see: Cerón-Camacho *et al.* (2006 ▶). For the synthesis, see: Bennett *et al.* (1982 ▶).
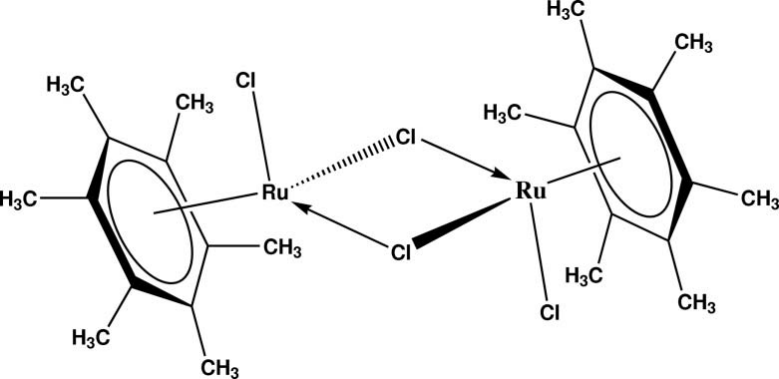


## Experimental

### 

#### Crystal data

[Ru_2_Cl_4_(C_12_H_18_)_2_]
*M* *_r_* = 668.47
Monoclinic, 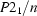

*a* = 8.9122 (15) Å
*b* = 8.5192 (15) Å
*c* = 16.642 (3) Å
β = 97.084 (3)°
*V* = 1253.9 (4) Å^3^
*Z* = 2
Mo *K*α radiation
μ = 1.64 mm^−1^
*T* = 298 K
0.23 × 0.09 × 0.05 mm

#### Data collection

Bruker SMART APEX CCD area-detector diffractometer
Absorption correction: multi-scan (*SADABS*; Bruker, 1999 ▶) *T*_min_ = 0.780, *T*_max_ = 0.924
10027 measured reflections
2297 independent reflections
1795 reflections with *I* > 2σ(*I*)
*R*_int_ = 0.065

#### Refinement

*R*[*F* ^2^ > 2σ(*F* ^2^)] = 0.033
*wR*(*F* ^2^) = 0.063
*S* = 0.90
2297 reflections
142 parameters
H-atom parameters constrained
Δρ_max_ = 0.48 e Å^−3^
Δρ_min_ = −0.38 e Å^−3^

### 

Data collection: *SMART* (Bruker, 1999 ▶); cell refinement: *SAINT* (Bruker, 1999 ▶); data reduction: *SAINT*; program(s) used to solve structure: *SHELXTL* (Sheldrick, 2008 ▶); program(s) used to refine structure: *SHELXTL*; molecular graphics: *SHELXTL*; software used to prepare material for publication: *SHELXTL*.

## Supplementary Material

Crystal structure: contains datablocks I, global. DOI: 10.1107/S1600536809041154/bt5080sup1.cif

Structure factors: contains datablocks I. DOI: 10.1107/S1600536809041154/bt5080Isup2.hkl

Additional supplementary materials:  crystallographic information; 3D view; checkCIF report

## Figures and Tables

**Fig. 1.**
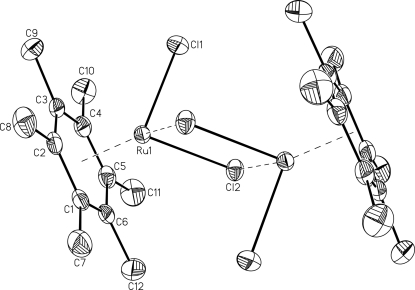
The molecular structure of the title compound with the atom numbering scheme. Displacement ellipsoids are drawn at the 40% probability level. H atoms omitted.

## supplementary crystallographic information

### Comment

For decades, arene ruthenium complexes have played an important role in organometallic chemistry as fundamental precursors for the synthesis of a plethora of species. This is particularly true for the synthesis of half sandwich ruthenium (II) complexes, species that have received considerable attention owing to their potential catalytic properties (Le Bozec  *et al.*, 1989), interesting anti-tumor and anti-carcinogenic activity (Leyva *et al.*, 2007) and most recently for their potential applications in chemical and biological sensors (Ryabov, *et al.*, 2001). Thus, given our continuous interest in the synthesis and catalytic applications of different ruthenium arene complexes (Cerón-Camacho *et al.*, 2006) we determined the crystal structure of the title compound.

The asymmetric unit of the title compound consists of a half molecule, which is completed with a symmetry operation of 1 - *x*, 1 - *y*, 1 - *z*. The complex exhibits a typical η^6^ - arene coordination of the hexamethyl fragment to the ruthenium centres which are  bridged by two chloro ligands. The coordination sphere of the Ru centres is completed by another chloro ligand. The aromatic rings and the Ru_2_Cl_2_ four-membered ring enclose a dihedral angle  of 55.85 (6)°.

### Experimental

The title compound was prepared according to the procedure reported by Bennett *et al.* (1982). The spectroscopic analysis agreed with that reported in the same reference.

### Refinement

H   atoms were placed in geometrically idealized positions with C-H = 0.96 Å and with *U*_iso_(H) = 1.2 U_eq_(C) and refined using the riding model. The methyl groups were allowed to rotate but not to tip.

### Crystal data

**Table:**

[Ru_2_Cl_4_(C_12_H_18_)_2_]	*F*(000) = 672
*M**_r_* = 668.47	*D*_x_ = 1.771 Mg m^−^^3^
Monoclinic, *P*2_1_/*n*	Mo *K*α radiation, λ = 0.71073 Å
Hall symbol: -P 2yn	Cell parameters from 4016 reflections
*a* = 8.9122 (15) Å	θ = 2.5–25.3°
*b* = 8.5192 (15) Å	µ = 1.64 mm^−^^1^
*c* = 16.642 (3) Å	*T* = 298 K
β = 97.084 (3)°	Prism, red
*V* = 1253.9 (4) Å^3^	0.23 × 0.09 × 0.05 mm
*Z* = 2	

### Data collection

**Table:**

Bruker SMART APEX CCD area-detector diffractometer	2297 independent reflections
Radiation source: fine-focus sealed tube	1795 reflections with *I* > 2σ(*I*)
graphite	*R*_int_ = 0.065
Detector resolution: 0.83 pixels mm^-1^	θ_max_ = 25.4°, θ_min_ = 2.5°
ω scans	*h* = −10→10
Absorption correction: multi-scan (*SADABS*; Bruker, 1999)	*k* = −10→10
*T*_min_ = 0.780, *T*_max_ = 0.924	*l* = −20→19
10027 measured reflections	

### Refinement

**Table:**

Refinement on *F*^2^	Primary atom site location: structure-invariant direct methods
Least-squares matrix: full	Secondary atom site location: difference Fourier map
*R*[*F*^2^ > 2σ(*F*^2^)] = 0.033	Hydrogen site location: inferred from neighbouring sites
*wR*(*F*^2^) = 0.063	H-atom parameters constrained
*S* = 0.90	*w* = 1/[σ^2^(*F*_o_^2^) + (0.0193*P*)^2^] where *P* = (*F*_o_^2^ + 2*F*_c_^2^)/3
2297 reflections	(Δ/σ)_max_ = 0.001
142 parameters	Δρ_max_ = 0.48 e Å^−^^3^
0 restraints	Δρ_min_ = −0.37 e Å^−^^3^

### Special details

**Table:**

Geometry. All e.s.d.'s (except the e.s.d. in the dihedral angle between two l.s. planes) are estimated using the full covariance matrix. The cell e.s.d.'s are taken into account individually in the estimation of e.s.d.'s in distances, angles and torsion angles; correlations between e.s.d.'s in cell parameters are only used when they are defined by crystal symmetry. An approximate (isotropic) treatment of cell e.s.d.'s is used for estimating e.s.d.'s involving l.s. planes.
Refinement. Refinement of *F*^2^ against ALL reflections. The weighted *R*-factor *wR* and goodness of fit *S* are based on *F*^2^, conventional *R*-factors *R* are based on *F*, with *F* set to zero for negative *F*^2^. The threshold expression of *F*^2^ > σ(*F*^2^) is used only for calculating *R*-factors(gt) *etc*. and is not relevant to the choice of reflections for refinement. *R*-factors based on *F*^2^ are statistically about twice as large as those based on *F*, and *R*- factors based on ALL data will be even larger.

### Fractional atomic coordinates and isotropic or equivalent isotropic displacement parameters (Å^2^)

**Table:**

	*x*	*y*	*z*	*U*_iso_*/*U*_eq_	
Ru1	0.37378 (4)	0.37262 (4)	0.55348 (2)	0.02300 (11)	
Cl1	0.24729 (13)	0.30866 (15)	0.42186 (7)	0.0382 (3)	
Cl2	0.61218 (11)	0.35592 (12)	0.49415 (6)	0.0297 (3)	
C1	0.4006 (5)	0.4464 (5)	0.6809 (2)	0.0316 (11)	
C2	0.2421 (5)	0.4425 (5)	0.6495 (2)	0.0293 (10)	
C3	0.1792 (5)	0.3056 (5)	0.6121 (2)	0.0281 (10)	
C4	0.2721 (5)	0.1702 (5)	0.6053 (3)	0.0312 (11)	
C5	0.4287 (5)	0.1741 (5)	0.6343 (3)	0.0341 (11)	
C6	0.4913 (5)	0.3142 (6)	0.6727 (2)	0.0325 (11)	
C7	0.4686 (6)	0.5943 (6)	0.7198 (3)	0.0524 (15)	
H7A	0.3892	0.6661	0.7287	0.063*	
H7B	0.5335	0.6420	0.6849	0.063*	
H7C	0.5263	0.5688	0.7707	0.063*	
C8	0.1465 (5)	0.5866 (5)	0.6552 (3)	0.0458 (13)	
H8A	0.1976	0.6762	0.6365	0.055*	
H8B	0.1297	0.6027	0.7105	0.055*	
H8C	0.0512	0.5729	0.6222	0.055*	
C9	0.0143 (5)	0.2997 (6)	0.5765 (3)	0.0451 (13)	
H9A	−0.0293	0.4025	0.5784	0.054*	
H9B	−0.0391	0.2280	0.6073	0.054*	
H9C	0.0068	0.2649	0.5213	0.054*	
C10	0.2020 (6)	0.0244 (5)	0.5645 (3)	0.0504 (14)	
H10A	0.2759	−0.0580	0.5677	0.060*	
H10B	0.1680	0.0470	0.5087	0.060*	
H10C	0.1177	−0.0082	0.5911	0.060*	
C11	0.5297 (6)	0.0355 (6)	0.6247 (3)	0.0563 (15)	
H11A	0.5450	−0.0223	0.6745	0.068*	
H11B	0.6254	0.0718	0.6110	0.068*	
H11C	0.4832	−0.0313	0.5823	0.068*	
C12	0.6585 (5)	0.3210 (6)	0.7036 (3)	0.0537 (15)	
H12A	0.6984	0.4212	0.6905	0.064*	
H12B	0.7106	0.2392	0.6786	0.064*	
H12C	0.6722	0.3069	0.7613	0.064*	

### Atomic displacement parameters (Å^2^)

**Table:**

	*U*^11^	*U*^22^	*U*^33^	*U*^12^	*U*^13^	*U*^23^
Ru1	0.02306 (18)	0.02546 (19)	0.02091 (18)	0.00141 (17)	0.00441 (13)	0.00103 (17)
Cl1	0.0363 (7)	0.0503 (8)	0.0268 (6)	−0.0021 (6)	−0.0011 (5)	−0.0047 (5)
Cl2	0.0296 (6)	0.0288 (6)	0.0324 (6)	0.0053 (5)	0.0104 (5)	0.0030 (5)
C1	0.036 (3)	0.042 (3)	0.018 (2)	−0.008 (2)	0.008 (2)	−0.002 (2)
C2	0.033 (3)	0.035 (3)	0.022 (2)	0.007 (2)	0.012 (2)	0.004 (2)
C3	0.027 (2)	0.036 (3)	0.023 (2)	0.000 (2)	0.0078 (19)	0.007 (2)
C4	0.038 (3)	0.029 (3)	0.028 (2)	−0.003 (2)	0.009 (2)	0.006 (2)
C5	0.040 (3)	0.034 (3)	0.030 (3)	0.004 (2)	0.011 (2)	0.010 (2)
C6	0.032 (3)	0.048 (3)	0.018 (2)	0.003 (2)	0.005 (2)	0.011 (2)
C7	0.055 (3)	0.064 (4)	0.040 (3)	−0.014 (3)	0.013 (3)	−0.017 (3)
C8	0.055 (3)	0.040 (3)	0.045 (3)	0.015 (3)	0.018 (3)	−0.006 (2)
C9	0.028 (3)	0.061 (3)	0.047 (3)	−0.005 (3)	0.004 (2)	0.004 (3)
C10	0.055 (3)	0.035 (3)	0.062 (4)	−0.005 (3)	0.010 (3)	−0.003 (3)
C11	0.061 (4)	0.048 (3)	0.061 (4)	0.018 (3)	0.012 (3)	0.010 (3)
C12	0.036 (3)	0.081 (4)	0.042 (3)	0.004 (3)	−0.003 (2)	0.014 (3)

### Geometric parameters (Å, °)

**Table:**

Ru1—C3	2.168 (4)	C5—C11	1.505 (6)
Ru1—C4	2.175 (4)	C6—C12	1.516 (6)
Ru1—C5	2.179 (4)	C7—H7A	0.9600
Ru1—C2	2.179 (4)	C7—H7B	0.9600
Ru1—C6	2.184 (4)	C7—H7C	0.9600
Ru1—C1	2.196 (4)	C8—H8A	0.9600
Ru1—Cl1	2.3993 (12)	C8—H8B	0.9600
Ru1—Cl2^i^	2.4528 (11)	C8—H8C	0.9600
Ru1—Cl2	2.4549 (11)	C9—H9A	0.9600
Cl2—Ru1^i^	2.4528 (11)	C9—H9B	0.9600
C1—C6	1.403 (6)	C9—H9C	0.9600
C1—C2	1.445 (6)	C10—H10A	0.9600
C1—C7	1.509 (6)	C10—H10B	0.9600
C2—C3	1.406 (6)	C10—H10C	0.9600
C2—C8	1.504 (5)	C11—H11A	0.9600
C3—C4	1.432 (6)	C11—H11B	0.9600
C3—C9	1.516 (6)	C11—H11C	0.9600
C4—C5	1.420 (6)	C12—H12A	0.9600
C4—C10	1.513 (6)	C12—H12B	0.9600
C5—C6	1.435 (6)	C12—H12C	0.9600
			
C3—Ru1—C4	38.51 (15)	C5—C4—C3	120.5 (4)
C3—Ru1—C5	69.46 (16)	C5—C4—C10	120.4 (4)
C4—Ru1—C5	38.06 (15)	C3—C4—C10	119.1 (4)
C3—Ru1—C2	37.75 (15)	C5—C4—Ru1	71.1 (2)
C4—Ru1—C2	68.75 (16)	C3—C4—Ru1	70.5 (2)
C5—Ru1—C2	81.93 (16)	C10—C4—Ru1	129.9 (3)
C3—Ru1—C6	81.49 (16)	C4—C5—C6	118.9 (4)
C4—Ru1—C6	68.64 (16)	C4—C5—C11	121.4 (4)
C5—Ru1—C6	38.39 (16)	C6—C5—C11	119.7 (4)
C2—Ru1—C6	68.72 (16)	C4—C5—Ru1	70.8 (2)
C3—Ru1—C1	68.85 (16)	C6—C5—Ru1	71.0 (2)
C4—Ru1—C1	81.11 (16)	C11—C5—Ru1	129.4 (3)
C5—Ru1—C1	68.71 (17)	C1—C6—C5	120.9 (4)
C2—Ru1—C1	38.56 (16)	C1—C6—C12	119.4 (4)
C6—Ru1—C1	37.35 (16)	C5—C6—C12	119.7 (4)
C3—Ru1—Cl1	92.23 (12)	C1—C6—Ru1	71.8 (2)
C4—Ru1—Cl1	90.57 (12)	C5—C6—Ru1	70.6 (2)
C5—Ru1—Cl1	115.57 (13)	C12—C6—Ru1	129.8 (3)
C2—Ru1—Cl1	119.79 (12)	C1—C7—H7A	109.5
C6—Ru1—Cl1	153.69 (13)	C1—C7—H7B	109.5
C1—Ru1—Cl1	158.35 (12)	H7A—C7—H7B	109.5
C3—Ru1—Cl2^i^	117.98 (12)	C1—C7—H7C	109.5
C4—Ru1—Cl2^i^	156.34 (12)	H7A—C7—H7C	109.5
C5—Ru1—Cl2^i^	156.20 (13)	H7B—C7—H7C	109.5
C2—Ru1—Cl2^i^	91.89 (12)	C2—C8—H8A	109.5
C6—Ru1—Cl2^i^	118.07 (13)	C2—C8—H8B	109.5
C1—Ru1—Cl2^i^	92.19 (12)	H8A—C8—H8B	109.5
Cl1—Ru1—Cl2^i^	87.48 (4)	C2—C8—H8C	109.5
C3—Ru1—Cl2	160.99 (12)	H8A—C8—H8C	109.5
C4—Ru1—Cl2	122.48 (12)	H8B—C8—H8C	109.5
C5—Ru1—Cl2	93.65 (12)	C3—C9—H9A	109.5
C2—Ru1—Cl2	151.86 (12)	C3—C9—H9B	109.5
C6—Ru1—Cl2	90.59 (12)	H9A—C9—H9B	109.5
C1—Ru1—Cl2	114.09 (12)	C3—C9—H9C	109.5
Cl1—Ru1—Cl2	87.26 (4)	H9A—C9—H9C	109.5
Cl2^i^—Ru1—Cl2	81.00 (4)	H9B—C9—H9C	109.5
Ru1^i^—Cl2—Ru1	99.00 (4)	C4—C10—H10A	109.5
C6—C1—C2	119.7 (4)	C4—C10—H10B	109.5
C6—C1—C7	120.4 (4)	H10A—C10—H10B	109.5
C2—C1—C7	119.8 (4)	C4—C10—H10C	109.5
C6—C1—Ru1	70.9 (2)	H10A—C10—H10C	109.5
C2—C1—Ru1	70.1 (2)	H10B—C10—H10C	109.5
C7—C1—Ru1	130.1 (3)	C5—C11—H11A	109.5
C3—C2—C1	119.8 (4)	C5—C11—H11B	109.5
C3—C2—C8	120.5 (4)	H11A—C11—H11B	109.5
C1—C2—C8	119.7 (4)	C5—C11—H11C	109.5
C3—C2—Ru1	70.7 (2)	H11A—C11—H11C	109.5
C1—C2—Ru1	71.3 (2)	H11B—C11—H11C	109.5
C8—C2—Ru1	129.0 (3)	C6—C12—H12A	109.5
C2—C3—C4	120.0 (4)	C6—C12—H12B	109.5
C2—C3—C9	121.0 (4)	H12A—C12—H12B	109.5
C4—C3—C9	119.0 (4)	C6—C12—H12C	109.5
C2—C3—Ru1	71.6 (2)	H12A—C12—H12C	109.5
C4—C3—Ru1	71.0 (2)	H12B—C12—H12C	109.5
C9—C3—Ru1	128.6 (3)		
			
C3—Ru1—Cl2—Ru1^i^	176.8 (4)	C9—C3—C4—C10	−1.3 (6)
C4—Ru1—Cl2—Ru1^i^	176.81 (14)	Ru1—C3—C4—C10	−125.6 (4)
C5—Ru1—Cl2—Ru1^i^	−156.66 (13)	C2—C3—C4—Ru1	−54.2 (3)
C2—Ru1—Cl2—Ru1^i^	−77.0 (2)	C9—C3—C4—Ru1	124.3 (4)
C6—Ru1—Cl2—Ru1^i^	−118.35 (13)	C3—Ru1—C4—C5	−133.7 (4)
C1—Ru1—Cl2—Ru1^i^	−88.37 (14)	C2—Ru1—C4—C5	−104.6 (3)
Cl1—Ru1—Cl2—Ru1^i^	87.88 (4)	C6—Ru1—C4—C5	−30.0 (3)
Cl2^i^—Ru1—Cl2—Ru1^i^	0.0	C1—Ru1—C4—C5	−66.6 (3)
C3—Ru1—C1—C6	104.2 (3)	Cl1—Ru1—C4—C5	133.4 (2)
C4—Ru1—C1—C6	66.3 (3)	Cl2^i^—Ru1—C4—C5	−141.6 (3)
C5—Ru1—C1—C6	28.9 (3)	Cl2—Ru1—C4—C5	46.3 (3)
C2—Ru1—C1—C6	133.2 (4)	C5—Ru1—C4—C3	133.7 (4)
Cl1—Ru1—C1—C6	134.7 (3)	C2—Ru1—C4—C3	29.1 (2)
Cl2^i^—Ru1—C1—C6	−136.6 (2)	C6—Ru1—C4—C3	103.7 (3)
Cl2—Ru1—C1—C6	−55.4 (3)	C1—Ru1—C4—C3	67.1 (3)
C3—Ru1—C1—C2	−29.0 (2)	Cl1—Ru1—C4—C3	−92.9 (2)
C4—Ru1—C1—C2	−66.9 (3)	Cl2^i^—Ru1—C4—C3	−7.8 (5)
C5—Ru1—C1—C2	−104.3 (3)	Cl2—Ru1—C4—C3	−180.0 (2)
C6—Ru1—C1—C2	−133.2 (4)	C3—Ru1—C4—C10	112.1 (5)
Cl1—Ru1—C1—C2	1.6 (5)	C5—Ru1—C4—C10	−114.2 (5)
Cl2^i^—Ru1—C1—C2	90.3 (2)	C2—Ru1—C4—C10	141.1 (5)
Cl2—Ru1—C1—C2	171.4 (2)	C6—Ru1—C4—C10	−144.3 (5)
C3—Ru1—C1—C7	−141.7 (5)	C1—Ru1—C4—C10	179.1 (4)
C4—Ru1—C1—C7	−179.6 (5)	Cl1—Ru1—C4—C10	19.2 (4)
C5—Ru1—C1—C7	143.0 (5)	Cl2^i^—Ru1—C4—C10	104.2 (4)
C2—Ru1—C1—C7	−112.7 (5)	Cl2—Ru1—C4—C10	−67.9 (4)
C6—Ru1—C1—C7	114.1 (5)	C3—C4—C5—C6	1.9 (6)
Cl1—Ru1—C1—C7	−111.1 (4)	C10—C4—C5—C6	−180.0 (4)
Cl2^i^—Ru1—C1—C7	−22.4 (4)	Ru1—C4—C5—C6	54.2 (4)
Cl2—Ru1—C1—C7	58.7 (5)	C3—C4—C5—C11	−177.4 (4)
C6—C1—C2—C3	0.9 (6)	C10—C4—C5—C11	0.7 (6)
C7—C1—C2—C3	179.0 (4)	Ru1—C4—C5—C11	−125.1 (4)
Ru1—C1—C2—C3	53.4 (3)	C3—C4—C5—Ru1	−52.3 (3)
C6—C1—C2—C8	−177.4 (4)	C10—C4—C5—Ru1	125.9 (4)
C7—C1—C2—C8	0.7 (6)	C3—Ru1—C5—C4	28.7 (3)
Ru1—C1—C2—C8	−124.9 (4)	C2—Ru1—C5—C4	65.6 (3)
C6—C1—C2—Ru1	−52.5 (3)	C6—Ru1—C5—C4	131.3 (4)
C7—C1—C2—Ru1	125.6 (4)	C1—Ru1—C5—C4	103.2 (3)
C4—Ru1—C2—C3	−29.6 (2)	Cl1—Ru1—C5—C4	−53.6 (3)
C5—Ru1—C2—C3	−66.7 (3)	Cl2^i^—Ru1—C5—C4	141.8 (3)
C6—Ru1—C2—C3	−104.1 (3)	Cl2—Ru1—C5—C4	−142.3 (2)
C1—Ru1—C2—C3	−132.4 (4)	C3—Ru1—C5—C6	−102.6 (3)
Cl1—Ru1—C2—C3	48.2 (3)	C4—Ru1—C5—C6	−131.3 (4)
Cl2^i^—Ru1—C2—C3	136.4 (2)	C2—Ru1—C5—C6	−65.7 (3)
Cl2—Ru1—C2—C3	−149.3 (2)	C1—Ru1—C5—C6	−28.1 (2)
C3—Ru1—C2—C1	132.4 (4)	Cl1—Ru1—C5—C6	175.0 (2)
C4—Ru1—C2—C1	102.8 (3)	Cl2^i^—Ru1—C5—C6	10.5 (5)
C5—Ru1—C2—C1	65.8 (3)	Cl2—Ru1—C5—C6	86.3 (2)
C6—Ru1—C2—C1	28.4 (3)	C3—Ru1—C5—C11	144.1 (5)
Cl1—Ru1—C2—C1	−179.3 (2)	C4—Ru1—C5—C11	115.4 (5)
Cl2^i^—Ru1—C2—C1	−91.2 (2)	C2—Ru1—C5—C11	−179.0 (5)
Cl2—Ru1—C2—C1	−16.8 (4)	C6—Ru1—C5—C11	−113.2 (5)
C3—Ru1—C2—C8	−114.0 (5)	C1—Ru1—C5—C11	−141.4 (5)
C4—Ru1—C2—C8	−143.7 (5)	Cl1—Ru1—C5—C11	61.8 (5)
C5—Ru1—C2—C8	179.3 (4)	Cl2^i^—Ru1—C5—C11	−102.8 (5)
C6—Ru1—C2—C8	141.9 (5)	Cl2—Ru1—C5—C11	−26.9 (4)
C1—Ru1—C2—C8	113.5 (5)	C2—C1—C6—C5	−0.6 (6)
Cl1—Ru1—C2—C8	−65.8 (4)	C7—C1—C6—C5	−178.7 (4)
Cl2^i^—Ru1—C2—C8	22.4 (4)	Ru1—C1—C6—C5	−52.8 (4)
Cl2—Ru1—C2—C8	96.7 (4)	C2—C1—C6—C12	178.2 (4)
C1—C2—C3—C4	0.2 (6)	C7—C1—C6—C12	0.1 (6)
C8—C2—C3—C4	178.5 (4)	Ru1—C1—C6—C12	126.1 (4)
Ru1—C2—C3—C4	53.9 (3)	C2—C1—C6—Ru1	52.2 (3)
C1—C2—C3—C9	−178.3 (4)	C7—C1—C6—Ru1	−125.9 (4)
C8—C2—C3—C9	0.0 (6)	C4—C5—C6—C1	−0.8 (6)
Ru1—C2—C3—C9	−124.5 (4)	C11—C5—C6—C1	178.5 (4)
C1—C2—C3—Ru1	−53.7 (3)	Ru1—C5—C6—C1	53.3 (4)
C8—C2—C3—Ru1	124.6 (4)	C4—C5—C6—C12	−179.6 (4)
C4—Ru1—C3—C2	132.3 (4)	C11—C5—C6—C12	−0.3 (6)
C5—Ru1—C3—C2	103.8 (3)	Ru1—C5—C6—C12	−125.5 (4)
C6—Ru1—C3—C2	66.0 (3)	C4—C5—C6—Ru1	−54.1 (4)
C1—Ru1—C3—C2	29.6 (2)	C11—C5—C6—Ru1	125.2 (4)
Cl1—Ru1—C3—C2	−139.6 (2)	C3—Ru1—C6—C1	−66.1 (3)
Cl2^i^—Ru1—C3—C2	−51.3 (3)	C4—Ru1—C6—C1	−103.8 (3)
Cl2—Ru1—C3—C2	132.3 (3)	C5—Ru1—C6—C1	−133.6 (4)
C5—Ru1—C3—C4	−28.4 (2)	C2—Ru1—C6—C1	−29.2 (2)
C2—Ru1—C3—C4	−132.3 (4)	Cl1—Ru1—C6—C1	−143.8 (3)
C6—Ru1—C3—C4	−66.2 (3)	Cl2^i^—Ru1—C6—C1	51.1 (3)
C1—Ru1—C3—C4	−102.7 (3)	Cl2—Ru1—C6—C1	131.2 (2)
Cl1—Ru1—C3—C4	88.1 (2)	C3—Ru1—C6—C5	67.5 (3)
Cl2^i^—Ru1—C3—C4	176.4 (2)	C4—Ru1—C6—C5	29.8 (2)
Cl2—Ru1—C3—C4	0.0 (5)	C2—Ru1—C6—C5	104.4 (3)
C4—Ru1—C3—C9	−112.4 (5)	C1—Ru1—C6—C5	133.6 (4)
C5—Ru1—C3—C9	−140.8 (4)	Cl1—Ru1—C6—C5	−10.1 (4)
C2—Ru1—C3—C9	115.4 (5)	Cl2^i^—Ru1—C6—C5	−175.2 (2)
C6—Ru1—C3—C9	−178.6 (4)	Cl2—Ru1—C6—C5	−95.1 (2)
C1—Ru1—C3—C9	144.9 (5)	C3—Ru1—C6—C12	−179.5 (5)
Cl1—Ru1—C3—C9	−24.3 (4)	C4—Ru1—C6—C12	142.7 (5)
Cl2^i^—Ru1—C3—C9	64.1 (4)	C5—Ru1—C6—C12	113.0 (6)
Cl2—Ru1—C3—C9	−112.3 (4)	C2—Ru1—C6—C12	−142.6 (5)
C2—C3—C4—C5	−1.7 (6)	C1—Ru1—C6—C12	−113.4 (6)
C9—C3—C4—C5	176.9 (4)	Cl1—Ru1—C6—C12	102.8 (5)
Ru1—C3—C4—C5	52.5 (4)	Cl2^i^—Ru1—C6—C12	−62.3 (5)
C2—C3—C4—C10	−179.8 (4)	Cl2—Ru1—C6—C12	17.8 (4)

Symmetry codes: (i) −*x*+1, −*y*+1, −*z*+1.
